# Improved antibacterial effects of alkali-transformed saponin from quinoa husks against halitosis-related bacteria

**DOI:** 10.1186/s12906-019-2455-2

**Published:** 2019-02-12

**Authors:** Xiaoyan Sun, Xiushi Yang, Peng Xue, Zhiguo Zhang, Guixing Ren

**Affiliations:** 1 College of Food Science and Engineering, Qilu University of Technology (Shandong Academy of Sciences), Jinan, 250353 China; 2 Institute of Crop Sciences, Chinese Academy of Agricultural Sciences, Beijing, 100081 China

**Keywords:** Quinoa, Halitosis, Alkali-transformation, Antibacterial activity, Antibacterial mechanism

## Abstract

**Background:**

Quinoa is a food crop native to the Andes. The process of dehulling quinoa can produce approximately 8–12% husk, which is often discarded because it contains bitter saponin. Saponin derived from quinoa has been reported to exhibit anti-inflammatory and antifungal activity. However, the antibacterial effects of quinoa saponin against halitosis-related bacteria are still unclear.

**Methods:**

In this study, quinoa saponin (QS) and alkali-transformed saponin (ATS) were separated by AB-2 resin to obtain QS-30, QS-80, ATS-30 and ATS-80. Halitosis-related bacteria included *Porphyromonas gingivalis* (*P. gingivalis*), *Clostridium perfringens* (*C. perfringens*) and *Fusobacterium nucleatum* (*F. nucleatum*). The MIC and MBC were determined using gradient dilutions in 96-well plates, and the saponins were identified by HPLC and mass spectrometry. The changes in membrane integrity were tested using a microplate reader, the membrane potential was tested by spectrofluorometry, and the morphological characteristics were examined using a transmission electron microscope to explore the antibacterial mechanisms.

**Results:**

Antibacterial assays indicated that QS-80 and ATS-80 showed inhibitory activity. In addition, ATS-80 exerted a stronger inhibitory effect than QS-80, especially against *Fusobacterium nucleatum*, with a lower minimum inhibitory concentration (31.3 μg/mL) and a lower minimum bactericidal concentration (125 μg/mL). ATS-80 destroyed the bacterial membrane structure, leading to bacterial death.

**Conclusions:**

Based on the excellent antibacterial activity and economic prospects of quinoa husk, ATS-80 could be used as an antibacterial agent to treat halitosis.

## Background

Halitosis is an unpleasant odor from the oral cavity caused by the catabolism of bacteria in the mouth and has an incidence of 22–50% for adults worldwide [1–3]. *Porphyromonas gingivalis* (*P. gingivalis*), *Clostridium perfringens* (*C. perfringens*), and *Fusobacterium nucleatum* (*F. nucleatum*) are considered the primary bacteria that induce halitosis [4]. Chemical ingredients, such as chlorhexidine, triclosan, and cetylpyridinium chloride, are often used to treat halitosis, but these chemicals may also induce side effects such as bacterial resistance and urticaria [5, 6]. In recent years, efforts have been made to identify an effective and safe antibiotic based on phytochemicals, such as essential oils, phenolic compounds, and saponins [7–9].

Quinoa (*Chenopodium quinoa* Willd) has been consumed for many years as a staple food in South America [10]. Due to the distinguished nutritional value and excellent tolerance to drought, cold, and saline conditions, quinoa has been widely planted on other continents, such as Europe, Africa, and Asia [11–13]. Saponin has been considered the most significant antinutritional component in the quinoa seed, with a content of 4.7–30.8 g/kg [14, 15]. The quinoa husk, which contains a higher saponin content than the grain, constitutes approximately 8–12% of the total crop and is separated from the grain during the dehulling process [16]. However, the quinoa husk is usually burned or discarded without being utilized. Recently, saponins from quinoa have been found to exhibit antibacterial activities against *Botrytis cinerea* and *Pomacea canaliculata* [17, 18], which suggests a possible use for the quinoa husk as an antibiotic.

Alkali transformation is a useful method to alter the chemical structures and biological activities of phytochemicals. This method has been successfully applied to quinoa saponin to improve its antifungal and molluscicidal activities [17, 18]. The enhanced activity might be related to the formation of more hydrophobic saponin derivatives, which could react more easily with cell membranes. However, the antibacterial activity of quinoa saponin against oral bacteria has rarely been reported, and the antibacterial mechanism is unclear. Therefore, in this study, the antibacterial effects of alkali-transformed saponin obtained from quinoa husk against *P. gingivalis*, *C. perfringens*, and *F. nucleatum* were investigated.

## Methods

### Material

The seed of quinoa (*Chenopodium quinoa* Willd) was provided by Mr. Wenjie Gao, general manager of Shanxi Yilong Quinoa Development Co. Ltd., Xinzhou, Shanxi, China in October 2016. Authentication of plant materials was established by professor Zhihua Zhu, crop specialist in Institute of Crop Sciences, Chinese Academy of Agricultural Sciences, Beijing, China. Voucher specimen (No. Jingli-1) is kept in the National Crop Gene Bank of Chinese Academy of Agricultural Sciences, Beijing, China.

High-performance liquid chromatography (HPLC) grade acetonitrile and methanol were obtained from Merck (Darmstadt, Germany). Deionized water (18 MVcm^− 1^) was prepared from distilled water through a Milli-Q system (Millipore, Bedford, MA, USA). The bacterial strains *Porphyromonas gingivalis* (ATCC 52066), *Clostridium perfringens* (ATCC 28188), and *Fusobacterium nucleatum* (ATCC 9533) were purchased from the Guangdong Microbiology Culture Center (Guangzhou, Guangdong, China).

### Extraction procedure

Quinoa was dehulled, and the husk was milled before passing through a 60-mesh sieve. The husk powder (500.00 g) was extracted with 4.0 L of methanol in a 50 °C water bath with agitation for 3 h. The solution was filtered and evaporated to obtain the saponin extract. This extract was dissolved in water and partitioned with ethyl acetate and n-butyl alcohol, sequentially. The n-butyl alcohol fraction was evaporated to obtain the crude quinoa saponin (QS, 48.68 g). QS (20 g) was dissolved in water and then loaded onto an AB-2 macroporous resin column. The column was eluted sequentially with deionized water, 30% methanol, and 80% methanol to obtain two saponin fractions, labeled QS-30 (0.88 g) and QS-80 (5.91 g).

### Alkali treatment

The alkali treatment of QS was performed according to a previously reported method with minor modifications [17]. Briefly, a 50 mg/mL QS (20 g) solution was prepared and mixed with 1 mol/L NaOH in a 95 °C water bath with agitation for 2.5 h. The mixture was cooled to room temperature, and 1 mol/L HCl was added to adjust the pH to 7. A dialysis bag with a molecular weight retention of 100 D was used to desalt the sample. After 12 h of dialysis, the retained solution was lyophilized to obtain alkali-transformed quinoa saponin (ATS, 18.95 g). ATS was dissolved and purified according to the procedure used for QS to obtain two fractions, labeled ATS-30 (0.54 g) and ATS-80 (6.12 g).

### Identification of the saponins

HPLC analysis was performed according to the method reported by Yao et al. [19], with some modifications. An Agilent SIL-20AT (Agilent, Santa Clara, California, USA) with an Alltima C18 column was used for the saponin analyses. The gradient elution was programmed using a mobile phase A consisting of deionized water and a mobile phase B consisting of acetonitrile. The elution program was as follows: 0 to 5 min, 15 to 24% B; 5 to 10 min, 24 to 30% B; 10 to 29 min, 30 to 55% B; 29 to 55 min, 55 to 90% B; 55 to 60 min, 90 to 15% B; 60 to 65 min, 15% B. The flow rate was 1 mL/min and the injection volume was 10 μL. A diode-array detector was used for detection, and the wavelengths of 210 nm and 254 nm were monitored.

The individual saponins were further identified using an LTQ Orbitrap mass spectrometer (Thermo Fisher Scientific Inc., Waltham, Massachusetts, USA), according to the method reported by Madl et al. [20]. The ESI source parameters were set as follows: heater temperature and capillary temperature were 350 °C, spray voltage was 3.5 KV, N_2_ flow was 35 psi, and aux gas flow was 10 psi. The ESI source was operated in the positive mode. Full MS scans were acquired in the range of 150–1500 m/z with a mass resolution of 30,000. The MS/MS and MS3 experiments were set as data-dependent scans. All analyses were performed in triplicate. The concentration of the individual saponins were evaluated using the calibration curve of oleanolic acid.

### Determination of minimum inhibitory concentration and minimum bactericidal concentration

*P. gingivalis*, *C. perfringen*, and *F. nucleatum* were cultured in CDC anaerobic blood agar base medium for 48 h at 37 °C in an anaerobic incubator (YQX-II, Shanghai, China). The minimum inhibitory concentration (MIC) and minimum bactericidal concentration (MBC) of the antibacterial agents were determined according to the methods reported by Wang et al. [21], with minor modifications. First, saponin samples were dissolved in dimethyl sulfoxide to a concentration of 10 mg/mL and then diluted with sterile GAM broth to achieve serial concentrations ranging from 2 mg/mL to 0.008 mg/mL. Diluted bacterial suspensions (100 μL) were added to 96-well microplates before the addition of different samples (100 μL). The final bacterial cell concentration in each well was 1 × 10^5^ colony-forming units per mL (CFU/mL). Agent-free broth and chlorhexidine were used as the blank and the positive control, respectively. The plates were incubated at 37 °C for 48 h. The MIC was defined as the lowest concentration of an antibacterial agent that inhibited bacterial growth, as indicated by the absence of turbidity. The MBC was determined by inoculating 10 μL of medium from each well of the MIC test that showed no turbidity onto CDC agar plates and incubating them at 37 °C for 48 h. The MBC values were defined as the lowest concentrations of antibacterial agents for which there was no bacterial growth on the plates. Each test was performed in triplicate.

### Integrity of the cell membrane

The integrity of the cell membrane was evaluated by measuring the release of bacterial nucleic acid and proteins into the cell suspension, according to the reported method [22]. Briefly, bacterial cells from 100 mL of a working culture were collected by centrifugation at 5000×g for 10 min. Cells were then washed three times and resuspended in 0.1 M phosphate-buffered saline (PBS, pH 7.4). The washed suspension (100 mL) was incubated at 37 °C in the presence of ATS-80 at different concentrations (control, MIC, and MBC). Every 4 h, the suspensions were collected and centrifuged at 6000×g for 5 min, and the supernatants were diluted with PBS. The absorption at 260 nm was measured by using a microplate reader (Spectramax Plus 384, Molecular Devices, Sunnyvale, CA, USA). Corrections were performed for the absorption of suspensions in PBS containing the same concentrations of ATS-80 after a 2 min reaction with the tested bacteria. QS at a concentration of 2 mg/mL was used as the negative control (NC). The untreated cells (control) were corrected with PBS only. In addition, suspensions were also collected to determine the protein concentrations using the Bradford method [23]. Untreated sample was used as a control. Each test was performed in triplicate.

### Membrane potential

The membrane potential (MP) was measured to evaluate the effect of quinoa saponins on the metabolic activity of bacterial cells [24]. Bacterial cells were incubated in GAM broth at 37 °C for 24 h. Cell suspensions (approximately 1 × 10^7^ CFU/mL) were incubated for 8 h with different concentrations of ATS-80 (control, MIC, and MBC) and then washed twice with PBS. The suspension was combined with rhodamine 123 and kept in the dark for 30 min. The cell suspension (100 μL) was transferred into 96-well microplates. The fluorescence was detected by a spectrofluorometer (SpectraMax M2e, Molecular Devices). The data are expressed as the mean fluorescence intensity (MFI). Each test was performed in triplicate.

### Visualization of cell damage with transmission electron microscope

Bacterial solutions (approximately 1 × 10^7^ CFU/ml) were combined with different concentrations of saponins (control, MIC, and MBC) and incubated for 24 h at 37 °C in an anaerobic incubator. The suspensions were harvested by centrifugation at 3000×g for 10 min. The bacteria were resuspended in 0.1 M phosphate buffer. Suspensions of *F. nucleatum* were treated with 0.5 mg/mL of CH_4_ for 10 h. After centrifugation at 3000×g for 10 min, the bacteria were harvested and embedded in 1.2% melted agar. The cell-agar blocks were fixed in 2.5% (*v*/v) glutaraldehyde in 0.1 M phosphate buffer (pH 7.2) for 90 min and washed three times in the same buffer for 10 min. The cell-agar blocks were postfixed for 2 h in 1% (*w*/*v*) osmium tetroxide, washed three times with sterile distilled water, and dehydrated using serial concentrations of ethanol (50, 60, 70, 80, 90, 95 and 100%) for 30 min each. After infiltration into a mixture (propylene oxide: resin = 1:3), the samples were embedded using the EPON-12 kit and polymerized at 60 °C for 72 h. The polymerized cell-agar blocks were sliced with an ultramicrotome (Reichert SuperNova; Leica, Wetzlar, Germany) to obtain thin sections (50–60 nm). Sections were mounted on grids and stained with uranyl acetate and Reynold’s lead citrate to enhance contrast. The morphological changes of the bacterial cells were then observed by transmission electron microscope, according to a previously reported method [25].

### Statistical analysis

All data are expressed as the mean ± standard deviation. Statistical analysis was performed in Microsoft Excel (Microsoft Corporation, Redmond, WA, USA) and IBM SPSS 19.0 (IBM Corporation, Armonk, NY, USA). One-way ANOVA with Dunnett’s post hoc test was used to determine the significant differences between group means at *P* < 0.05 and *P* < 0.01.

## Results

### Antibacterial activity of different saponin fractions

The MIC and MBC values of the different quinoa saponin fractions and the positive control chlorhexidine are presented in Table 1. QS showed no inhibitory effects against the bacteria, even at a concentration of 2000 μg/mL. However, ATS exhibited inhibitory effects on the bacteria at concentrations of 250 and 500 μg/mL. After separation by resin, neither QS-30 nor ATS-30 could inhibit the growth of the bacteria. QS-80 showed antibacterial activity, but it was not stronger than that of ATS-80, which could inhibit the growth of *C. perfringen* and *F. nucleatum* at a concentration of 31.3 μg/mL. Among the three bacteria, *F. nucleatum* was the most susceptible to ATS-80, with the lowest MIC and MBC values of 31.3 μg/mL and 125 μg/mL, respectively.

**Table 1:** MIC and MBC values of saponin fractions against the three halitosis bacteria (μg/mL)

Sample	*P. gingivalis*		*C. perfringens*		*F. nucleatum*	
	MIC	MBC	MIC	MBC	MIC	MBC
QS	> 2000	> 2000	> 2000	> 2000	> 2000	> 2000
AQS	500	2000	250	2000	250	2000
QS-30	> 2000	> 2000	> 2000	> 2000	> 2000	> 2000
ATS-30	> 2000	> 2000	> 2000	> 2000	> 2000	> 2000
QS-80	1000	> 2000	1000	2000	1000	> 2000
ATS-80	62.5	250	31.3	250	31.3	125
Chlorhexidine	16.0	125	16.0	125	8.0	62.5

*MIC* minimum inhibitory concentration, *MBC* minimum bactericidal concentration, *QS* quinoa saponin, *ATS* alkali-transformed quinoa saponin, QS-30 and ATS-30 were the 30% methanol-eluted fractions, and QS-80 and ATS-80 were the 80% methanol-eluted fractions from QS and ATS, respectively

### Identification of individual saponins in QS-80 and ATS-80

**Fig. 1:**
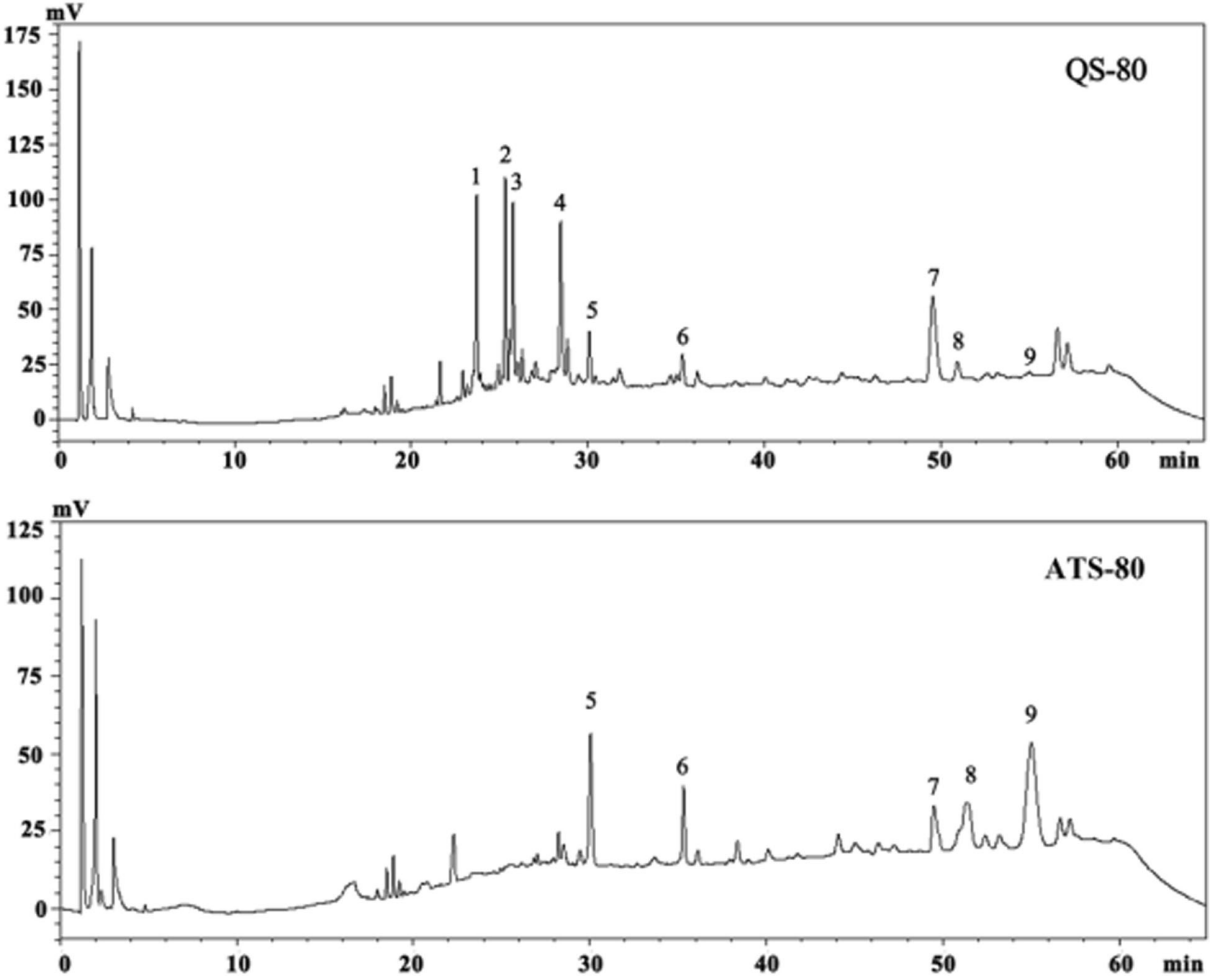
HPLC chromatograms of QS-80 and ATS-80. QS-80 and ATS-80 were the 80% methanol-eluted fractions from quinoa saponin and alkali-transformed quinoa saponin, respectively

The compositions of QS-80 and ATS-80 are shown in Fig. 1, and nine saponins were identified in QS-80. According to the mass data and literature information, the formulae of these saponins were determined [19, 26]. Six of these compounds (peaks 1, 3, 4 and 5) were also found in quinoa seed, as reported by Yao et al. [19], and the others were reported by Fumie et al. [27]. The contents of each saponin in the two fractions are shown in Table 2.

**Table 2:** Average concentration of saponins in QS-80 and ATS-80

Peak	Possible compound	Formula	[M + H]^+^	QS-80 (mg/g)	ATS-80 (mg/g)
1	3-O-β-D-glucopyranosyl-(1 → 3)-α-L-arabinopyranosyl phytolaccagenic acid 28-O-β-D-glucopyranosyl ester	C_48_H_76_O_20_	973	66.26 ± 1.98^a^	nd
2	3-O-β-D-xylopyranosyl-(1 → 3)-β-D-glucuronopyranosyl oleanolic acid 28-O-β-D-glucopyranosyl ester	C_47_H_74_O_18_	927	73.34 ± 2.32^a^	nd
3	3-O-β-D-glucopyranosyl-(1 → 3)-α-L-arabinopyranosyl hederagenin acid 28-O-β- D-glucopyranosyl ester	C_47_H_76_O_18_	929	64.07 ± 2.68^a^	nd
4	3-O-β-D-xylopyranosyl-(1 → 3)-β-D-glucuronopyranosyl hederagenin acid 28-O-β- D-glucopyranosyl ester	C_47_H_74_O_19_	943	80.83 ± 1.74^a^	nd
5	3-O-α-L-arabinopyranosyl phytolaccagenic acid 28-O-β-D-glucuronopyranosyl ester	C_42_H_66_O_15_	811	18.43 ± 0.87^b^	71.05 ± 1.75^a^
6	3-O-α-L-arabinopyranosyl hederagenin acid 28-O-β-D-glucopyranosyl ester	C_41_H_66_O_13_	767	10.21 ± 0.55^b^	23.22 ± 0.51^a^
7	3-O-β-D-glucuronopyranosyl oleanolic acid 28-O-β-D-glucopyranosyl ester	C_42_H_66_O_14_	795	159.47 ± 3.62^a^	80.96 ± 3.25^b^
8	3-O-H hederagenin acid 28-O-H	C_30_H_48_O_4_	473	17.86 ± 0.49^b^	83.06 ± 2.90^a^
9	3-O-H oleanolic acid 28-O-H	C_30_H_48_O_3_	457	2.65 ± 0.08^b^	83.58 ± 3.89^a^
Total				493.12 ± 4.57^a^	341.87 ± 4.66^b^

QS-80 and ATS-80 were the 80% methanol-eluted fractions from quinoa saponin and alkali-transformed quinoa saponin, respectively; nd: not detected. Data in each row that are followed by different letters are significantly different (*P* < 0.05)

**Fig. 2:**
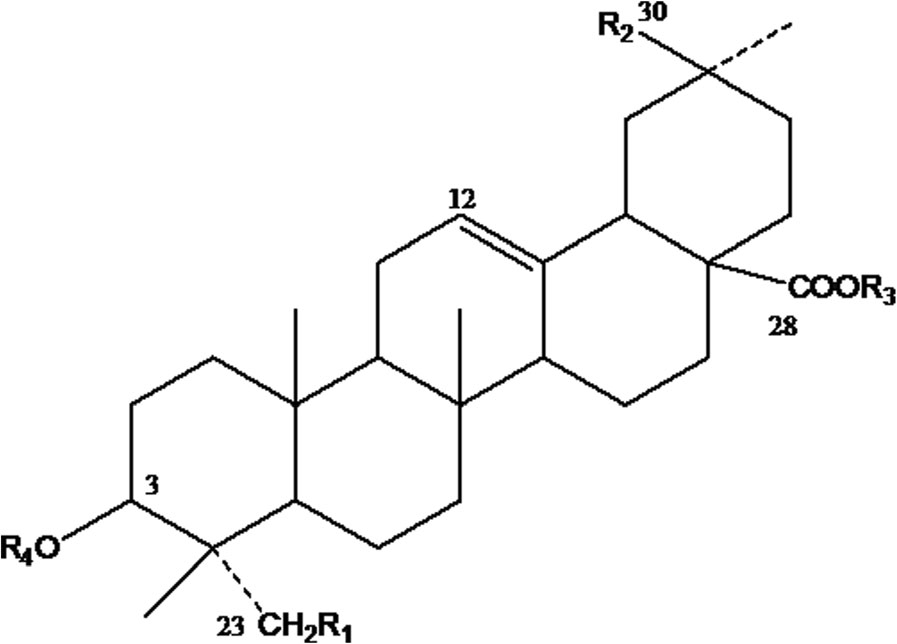
The core structure of QS-80 and ATS-80. QS-80 and ATS-80 were the 80% methanol-eluted fractions from quinoa saponin and alkali-transformed quinoa saponin, respectively

The contents of individual saponins in QS-80 largely differed from those in ATS-80. The total content of saponins in QS-80 was 493.12 mg/g, which was not significantly different from that of ATS-80 (472.37 mg/g). The contents of peaks 1, 2, 4, and 7 in QS-80 were 66.26, 73.34, 80.83, and 159.47 mg/g, respectively, which were all significantly (*P* < 0.05) higher than those in ATS-80. Peaks 1, 2, 3 and 4 could not be detected in ATS-80. However, the contents of peaks 5, 6, 7, 8, and 9 in ATS-80 were 71.05, 23.22, 80.96, 83.06 and 83.58 mg/g, respectively, which were remarkably higher (*P* < 0.05) than those in QS-80. The content variation of saponins could be observed from the heights and widths of the peaks in Fig. 1. The structure of the saponin monomer is shown in Fig. 2. The R-group in the structure of each saponin monomer is shown in Table 3.

**Table 3:** The R-group in the structure of each saponin monomer

Peak	R_1_	R_2_	R_3_	R_4_
1	-OH	COOCH3	-Glc	-Ara-Glc
2	-H	CH3	-Glc	-GlcA-Xyl
3	-OH	CH3	-Glc	-Ara-Glc
4	-OH	CH3	-Glc	-GlcA-Xyl
5	-OH	COOCH3	-Glc	-Ara
6	-OH	CH3	-Glc	-Ara
7	-H	CH3	-Glc	-GlcA
8	-OH	CH3	-H	-H
9	-H	CH3	-H	-H

Ara: α-L-arabinopyranosyl; Glc: β-D-glucopyranosyl; GlcA: β-D-glucuronopyranosyl; Xyl: β-D-xylopyranosyl

In this study, the saponins at peaks 1, 2, 3, and 4 contained three glycosyls and could be considered to be highly polar saponins. These saponins might lose sugar and convert to less polar saponins. The saponins from peak 1 can lose one Glc to convert to the saponin from peak 5; the saponin from peak 2 can lose one Xyl to convert to the saponin from peak 7 or can lose the side chains -GlcA-Xyl and Glc to convert to the saponin from peak 9; the saponin from peak 3 can lose one Glc to convert to the saponin from peak 6 or can lose the side chains -Ara-Glc and Glc to convert to the saponin from peak 8; and the saponin from peak 4 can lose the side chains -GlcA-Xyl and Glc to convert to the saponin from peak 8. The total content of all of the less polar saponins in ATS-80 was approximately 1.5 times that in QS. In addition, the total content of peaks 5, 6, 8, and 9 in ATS-80 was more than 6 times that in QS-80.

### Integrity of the cell membrane

**Fig. 3:**
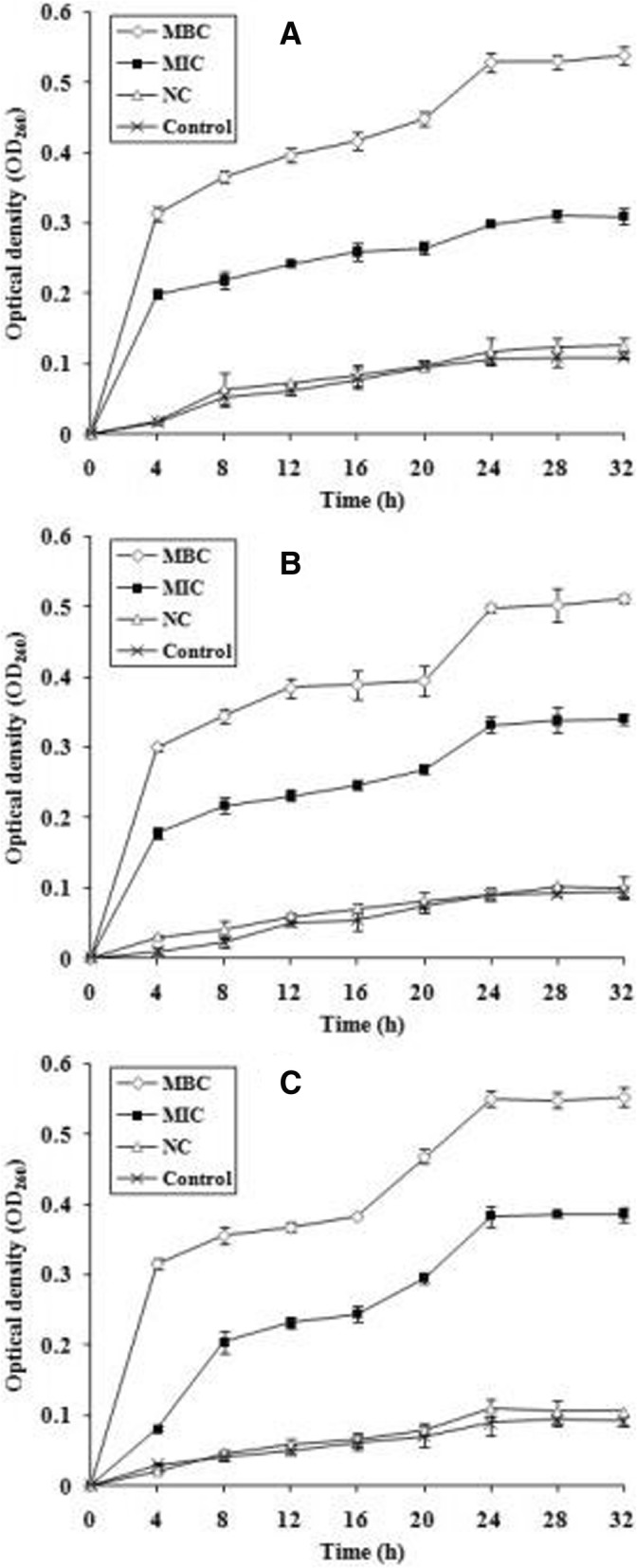
Release of 260-nm absorbing material from *P. gingivalis* (**a**), *C. perfringens* (**b**), and *F. nucleatum* (**c**) treated with ATS-80 for 32 h. ATS-80, 80% methanol-eluted fractions from alkali-treated quinoa saponin; MBC, minimum bactericide concentration; MIC, minimum inhibition concentration; NC, negative control (quinoa saponin)

The results of the optimal density at 260 nm (OD_260nm_), which indicated that nucleic acids were released into the cell suspension, are shown in Fig. 3.

The optical density at 260 nm of these bacteria increased in the presence of ATS-80 at the MIC and MBC when compared with the control. This result clearly indicates that ATS-80 induces the leakage of nucleic acids into the cell suspension. However, there was almost no difference between the OD_260_ value of the NC and the control. The OD_260_ values of the three bacteria treated with ATS-80 steadily increased from 4 h to 24 h. After that, the values remained almost stable for the next eight hours. Table 4 shows the OD_260_ values of the three bacteria after 24 h treatment, as well as the protein concentrations.

**Table 4:** Effect of ATS-80 on the nucleic acid and protein release of tested bacteria

Group	Nucleic acid (OD_260nm_)			Protein (μg/mL)		
	*P. gingivalis*	*C. perfringens*	*F. nucleatum*	*P. gingivalis*	*C. perfringens*	*F. nucleatum*
MBC	0.528 ± 0.013^a^	0.498 ± 0.006^a^	0.551 ± 0.012^a^	157.84 ± 11.29^a^	185.68 ± 12.54^a^	207.84 ± 15.52^a^
MIC	0.298 ± 0.004^b^	0.332 ± 0.011^b^	0.383 ± 0.015^b^	54.59 ± 4.37^b^	108.92 ± 8.83^b^	118.92 ± 9.85^b^
NC	0.117 ± 0.020^c^	0.091 ± 0.009^c^	0.110 ± 0.012^c^	10.15 ± 0.88^c^	10.33 ± 1.45^c^	11.56 ± 1.87^c^
Control	0.107 ± 0.008^c^	0.089 ± 0.006^c^	0.089 ± 0.018^c^	9.68 ± 0.96^c^	10.14 ± 0.75^c^	9.73 ± 1.06^c^

ATS-80, 80% methanol-eluted fractions from alkali-transformed quinoa saponin. Data in each column that are followed by different letters are significantly different (*P* < 0.05)

The results indicated that the release of nucleic acid and protein increased with increasing concentrations of ATS-80. For *F. nucleatum*, the OD_260nm_ value and the released protein content for bacteria treated with ATS-80 at the MIC increased 4.30 and 12.22 times, respectively, compared with those of the control; when treated with ATS-80 at the MBC, they increased by 6.19 and 21.36 times, respectively. Similar results were also observed for the other two bacteria.

### Membrane potential

**Fig. 4:**
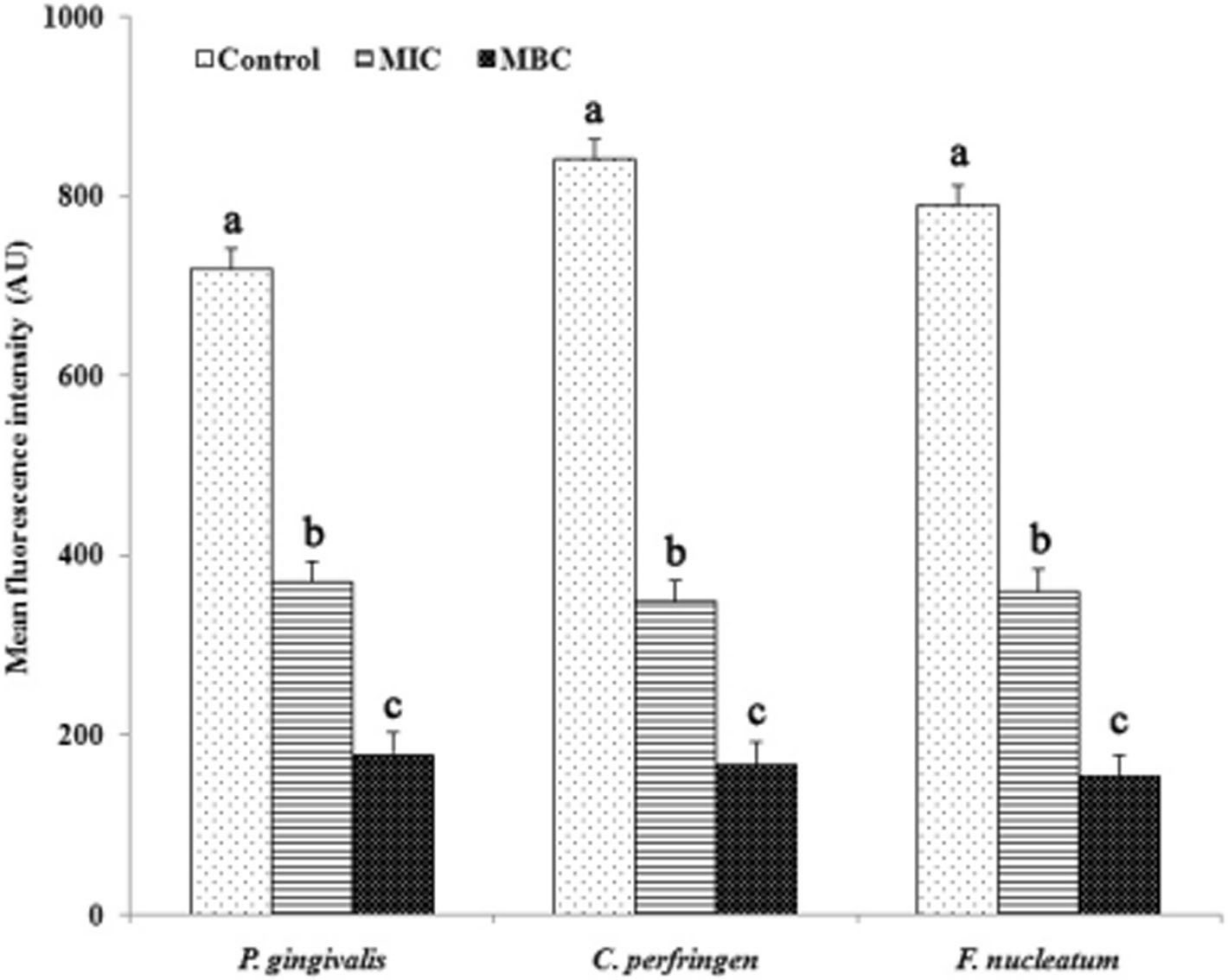
Membrane potentials of *P. gingivalis*, *C. perfringens*, and *F. nucleatum* treated with ATS-80. ATS-80, 80% methanol-eluted fractions from alkali-treated quinoa saponin; MIC: minimum inhibition concentration; MBC: minimum bactericide concentration

The membrane potential results are presented in Fig. 4. After the addition of ATS-80 at the MIC, the MFI value was reduced by 48.40, 58.44, and 54.33% for *P. gingivalis*, *C. perfringens*, and *F. nucleatum*, respectively, compared with the control. Furthermore, ATS-80 at the MBC induced 75.07, 80.13, and 80.38% decreases in the MFI value for *P. gingivalis*, *C. perfringens*, and *F. nucleatum*, respectively.

### Morphological alterations

**Fig. 5:**
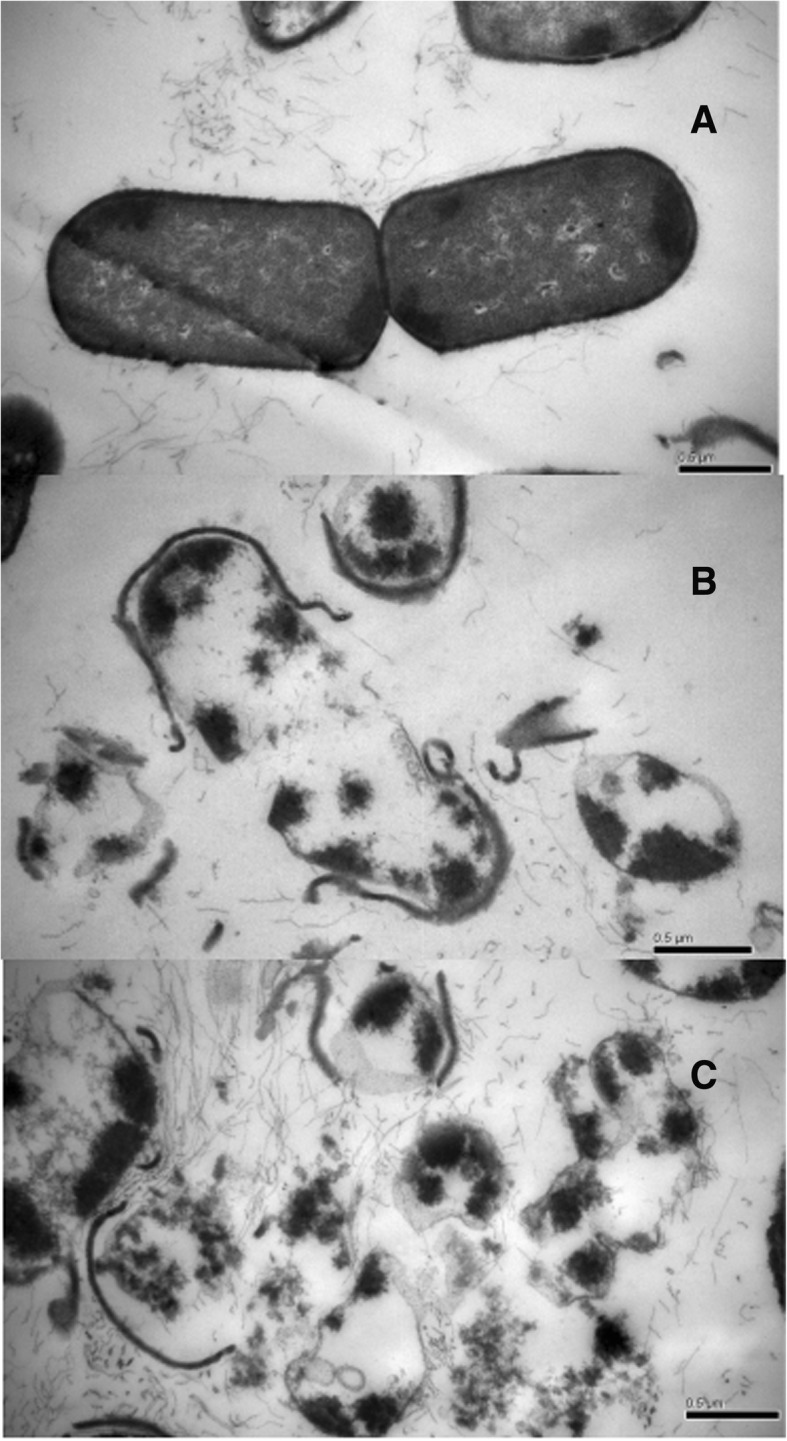
The morphological alterations of *F. nucleatum* cells treated with different concentrations of ATS-80. ATS-80, 80% methanol-eluted fractions from alkali-treated quinoa saponin; **a**, control; **b**, minimum inhibition concentration; **c**, minimum bactericide concentration

As shown in Fig. 5a, *F. nucleatum* cells in the control group had typical cell structures, with an intact cell wall and homogeneous intracellular constituents. However, cells treated with ATS-80 at the MIC showed great morphological alterations (Fig. 5b). An incomplete cell shape was observed. The cell wall and membrane were disintegrated, with nucleic acid and protein coagulating and aggregating near the cell membrane. Cells treated with ATS-80 at the MBC exhibited a deformed shape, showing rupture of the membranes with nucleic acid and protein leaching out of the cell (Fig. 5c).

## Discussion

Combined, the results of the antibacterial activities of different saponin fractions and the identification of individual saponins in QS-80 and ATS-80 suggest that alkali transformation could improve the antibacterial effects of quinoa saponin against oral bacteria. Stuardo and Martín [18] reported that NaOH treatment could enhance the inhibitory activity of quinoa saponin against *Botrytis cinerea.* This enhancement was attributed to the formation of more hydrophobic saponin derivatives, which could more easily react with the bacterial cell membrane. In this study, the 80% methanol-eluted fractions (QS-80 and ATS-80), which contained saponins with lower polarities, showed higher antibacterial activities than the corresponding 30% methanol-eluted fractions. However, the profile of alkali-transformed quinoa saponins and the structural changes of the saponins were still not well elucidated.

To explore the superior antibacterial activity of ATS-80, we examined the chemical structure changes. The polarity of saponins are positively correlated with the number of sugar moieties. Highly polar saponins that have more sugar moieties could be more easily dissolved in water and dissociated from the reversed-phase separation. It has been reported that highly polar saponins in *Panax ginseng* and American ginseng could be converted to less polar saponins by deglycosylation with heat treatment [9, 21]. The less polar ginsenosides exhibited higher antibacterial activities than the highly polar ones. They had better lipophilic properties and bound to bacterial cell membranes, thereby affecting the physiological activities of the cells. It might be inferred that less polar saponins, especially the saponins from peaks 8 and 9, played key roles in the superior antibacterial activity of ATS-80. However, further antibacterial assays should be performed on individual saponins after the separation and purification of these saponins in the future.

The integrity of the cytoplasmic membrane is essential for cell growth. Detecting the leakage of cell constituents, such as nucleic acid and proteins, can reveal the integrity of the cell membrane [28, 29]. To examine whether the less polar quinoa saponins could react with bacterial cell membranes, the effect of ATS-80 on the integrity of the cell membrane was studied. These results clearly indicated that the integrity of the bacterial cell membranes were damaged after exposure to ATS-80, which led to the loss of intracellular constituents. The leakage of nucleic acids and proteins revealed the malfunction of the cell membrane, which will lead to cell death. However, as the negative control, QS showed no effect on the integrity of the cell membrane.

The MP of normal bacteria is generated by a difference in the concentration of ions on opposite sides of the cell membrane, and it plays an important role in bacterial physiology. MP is an element of the proton motive force, and it is involved in the generation of ATP [30]. That is, the alteration of the MP in bacteria could affect cell metabolic activity [22]. In the present study, the bacterial MP was directly correlated with MFI. Therefore, based on the combined fluorescence intensity, ATS-80 could significantly reduce the MP of halitosis related bacteria. The reduction in MP revealed cell membrane depolarization, which could eventually lead to irregular metabolic activity and bacterial death [24].

The morphological alterations could more intuitive reflect the principle of action for less polar saponins. The morphological changes observed in bacteria treated with different concentration of ATS-80 further suggest that ATS-80 can affect the integrity of bacterial cell membranes and induce the loss of intracellular constituents. In *F. nucleatum,* when treated with the MBC of ATS-80, extracellular protoplasm, pore formation, and the disintegration of the cells were observed, all of which are irreversible changes. Less polar saponins can cause damage by acting on hydrophobic structures of the cytoplasmic membrane or cell wall [31]; therefore, further work is required to fully understand the mechanisms involved.

## Conclusions

This work clearly indicated that alkali-transformed saponins from quinoa husk, especially ATS-80, showed higher antibacterial activities against three halitosis-related bacteria than the primitive quinoa saponins. One major reason for this result was that the less polar saponins in ATS-80 were able to more easily interact with the bacterial cell membranes and damage the integrity of the membranes, as well as decrease the membrane potential. Based on the superior antibacterial activity and economic aspects of quinoa husk, ATS-80 might be applied as an antibacterial agent to treat halitosis.

## Abbreviations

AQS: Alkali-transformed Quinoa Saponin
Ara: α-L-arabinopyranosyl
ATS-30 and ATS-80: the 30 and 80% methanol-eluted fractions from ATS, respectively
*C. perfringens*: *Clostridium perfringens*
CFU/mL: colony-forming units per mL
CLSI: Clinical and Laboratory Standards Institute
*F. nucleatum*: *Fusobacterium nucleatum*
Glc: β-D-glucopyranosyl
GlcA: β-D-glucuronopyranosyl
HPLC: High-performance liquid chromatography
MBC: minimum bactericidal concentration
MFI: mean fluorescence intensity;
MIC: minimum inhibitory concentration
MP: membrane potential
NC: negative control
OD_260nm_: optimal density at 260 nm
*P. gingivalis*: *Porphyromonas gingivalis*
PBS: phosphate-buffered saline
QS: Quinoa Saponins
QS-30 and QS-80: the 30 and 80% methanol-eluted fractions from QS, respectively
Xyl: β-D-xylopyranosyl
